# From dinosaurs to birds: a tail of evolution

**DOI:** 10.1186/2041-9139-5-25

**Published:** 2014-07-29

**Authors:** Dana J Rashid, Susan C Chapman, Hans CE Larsson, Chris L Organ, Anne-Gaelle Bebin, Christa S Merzdorf, Roger Bradley, John R Horner

**Affiliations:** 1 Museum of the Rockies, Montana State University, 600 West Kagy Blvd, Bozeman, MT 59717, USA; 2 Department of Biological Sciences, Clemson University, 340 Long Hall, Clemson, SC 29634, USA; 3 Redpath Museum, McGill University, 859 Sherbrooke Street W., Montreal, Quebec H3A 0C4, Canada; 4 Department of Earth Sciences, Montana State University, 226 Traphagen Hall, Bozeman, MT 59717, USA; 5 Current address: Vaccine and Gene Therapy FL, 9801 Discovery Way, Port Lucie, FL 34987, USA; 6 Department of Cell Biology & Neuroscience, Montana State University, 513 Leon Johnson Hall, Bozeman, MT 59717, USA

**Keywords:** *Archaeopteryx*, Avian, Bird evolution, *Confuciusornis*, Dinosaur, *Jeholornis*, *Sapeornis*, Somitogenesis, Tail

## Abstract

A particularly critical event in avian evolution was the transition from long- to short-tailed birds. Primitive bird tails underwent significant alteration, most notably reduction of the number of caudal vertebrae and fusion of the distal caudal vertebrae into an ossified pygostyle. These changes, among others, occurred over a very short evolutionary interval, which brings into focus the underlying mechanisms behind those changes. Despite the wealth of studies delving into avian evolution, virtually nothing is understood about the genetic and developmental events responsible for the emergence of short, fused tails. In this review, we summarize the current understanding of the signaling pathways and morphological events that contribute to tail extension and termination and examine how mutations affecting the genes that control these pathways might influence the evolution of the avian tail. To generate a list of candidate genes that may have been modulated in the transition to short-tailed birds, we analyzed a comprehensive set of mouse mutants. Interestingly, a prevalent pleiotropic effect of mutations that cause fused caudal vertebral bodies (as in the pygostyles of birds) is tail truncation. We identified 23 mutations in this class, and these were primarily restricted to genes involved in axial extension. At least half of the mutations that cause short, fused tails lie in the Notch/Wnt pathway of somite boundary formation or differentiation, leading to changes in somite number or size. Several of the mutations also cause additional bone fusions in the trunk skeleton, reminiscent of those observed in primitive and modern birds. All of our findings were correlated to the fossil record. An open question is whether the relatively sudden appearance of short-tailed birds in the fossil record could be accounted for, at least in part, by the pleiotropic effects generated by a relatively small number of mutational events.

## Review

### Introduction

Tails of extant birds are an evolutionary novelty. They are critical for powered flight, ensure reproductive success by attracting mates, and safeguard relatives by communicating warning signals. Extant bird tails consist proximally of a small series of unfused caudal vertebrae with a high range of motion. These articulate to a distal rod-like pygostyle, composed of several fused caudal vertebrae, which supports the retricial bulb and associated muscles and feathers for controlling tail fan and contour shape. This specialized tail is present throughout the entire diversity of living birds, albeit with many modifications for clade-specific behaviors. A well-sampled fossil record documents the evolutionary transformation from the ancestral long 'reptilian' tail to the short, distally fused tail [1,2]. Yet, the specific developmental mechanisms that facilitated this dramatic anatomical change are unknown. This gap in knowledge constitutes a rich vein for future research, one that would benefit from our current understanding of axial developmental mechanisms.

The phenotypic changes that arose in the transition from ancestral long-tailed to short-tailed birds were manifested from changes during embryonic development, indicating that the study of mutations in embryonic models is essential to elucidating the mechanism of tail shortening. Mutations in key developmental genes and/or their regulation can cause multiple changes in morphology. Indeed, pleiotropic effects are observed in the vertebrate axial skeleton for a number of mutations [3]. Given the multiple phenotypes that can arise with single mutations, can the perceived sudden appearance of short-tailed birds be due to a lack of intermediate specimens in the fossil record, or from a very limited number of mutations that caused significant alterations to the primitive bird skeleton in a relatively short period of time? With reference to the fossil record, we first review the evolutionary history and early skeletal development of the bird tail, and discuss the developmental mechanisms involved in axial extension and termination. We then present a comprehensive survey of mouse tail mutants with the purpose of examining conserved patterns of mutation and likely candidates within the tail gene regulatory network that may have played major roles in reducing the bird tail. These paleontological, genetic, and developmental analyses are applied to the critical juncture in bird evolution at which tails were truncated and flight was greatly enhanced, in the late Jurassic to Early Cretaceous transition of primitive long-tailed to short-tailed birds.

## Background

**Figure 1:**
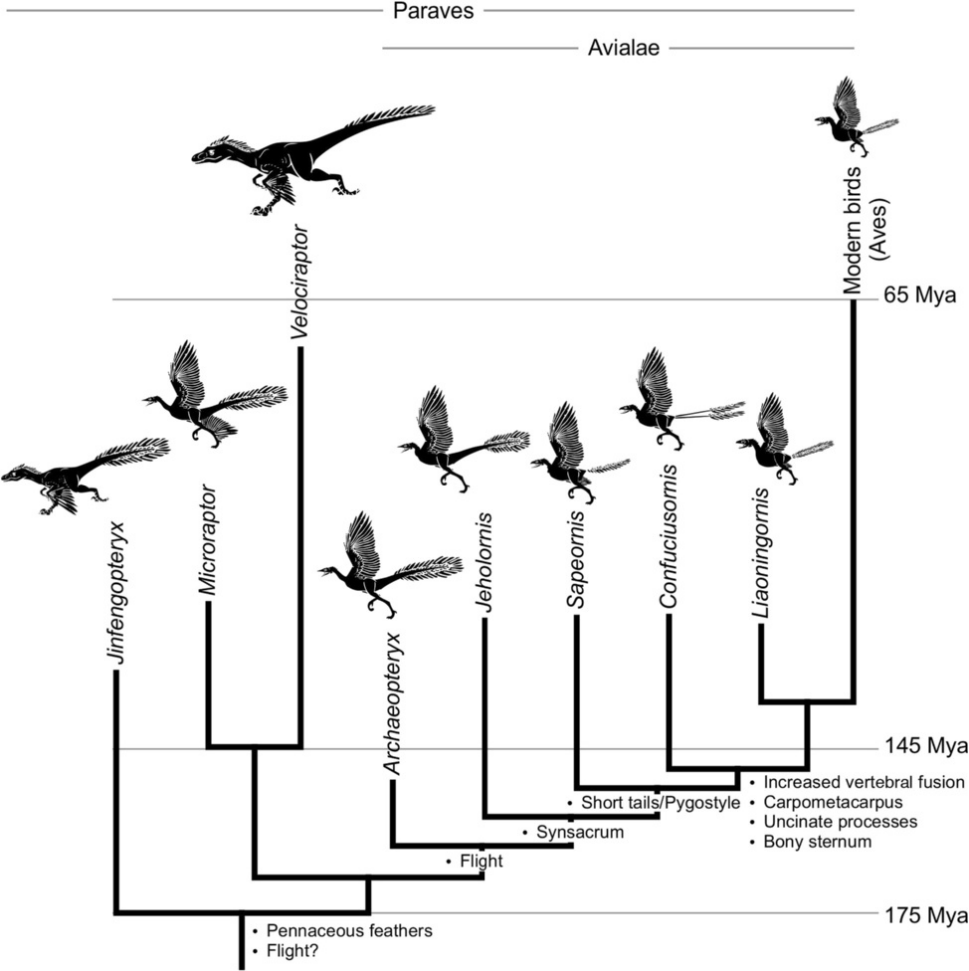
**Evolutionary tree of Paraves showing important evolutionary changes.** Although several other groups of dinosaurs evolved a pygostyle (fused posterior tail vertebrae) independently, note that the first birds had long tails and that the fossil record documents a short temporal duration of both long- and short-tailed birds followed thereafter exclusively by birds with truncated, distally fused tails.

The origin of the derived bird tail occurred over a remarkably short evolutionary interval, as evidenced by the short-lived co-occurrence of both long- and short-tailed birds in equivalent spatio-temporal fossil formations (Figure 1). Nearly all non-avian theropod dinosaurs sported long, 'reptilian' tails. These taxa were bipedal, so the tail likely had a counterbalance function. It also had robust transverse processes on the proximal caudal vertebrae that would have served as attachment sites for the large caudofemoralis muscles that were the primary hind limb retractors [4]. The oldest known bird, *Archaeopteryx*, dated to 150 million years ago, defines the clade Aves [5-7] or Avialae [8]. Its fully formed flight feathers, elongated wings, and evidence of capable powered flight, all ally *Archaeopteryx* with birds [9,10]. Yet, the presence of teeth, clawed and unfused fingers, and an elongated, bony tail are characteristics shared with non-avian theropod dinosaurs. Paravians, including *Archaeopteryx*, are characterized by long tails [11,12], some fusion of synsacral vertebrae, and varying flight capability (Figure 1). Most deinonychosaurians had between 20 and 30 caudal vertebrae. Oviraptorosaurs, probably the immediate outgroup to Paraves, had relatively shorter tails. These shorter tails were due not just to a modest decrease in the number of caudal vertebrae relative to other non-avian theropods, but more generally to a reduction in individual lengths of the more distal caudals [13]. Interestingly, several oviraptorosaurs have been documented to have the distal caudal vertebrae co-ossified into a pygostyle-like structure that braced a fan-like arrangement of retrices [13-16]. Another more prominent independent reduction of tail length occurred in *Epidexipteryx*, a Mid- or Late-Jurassic maniraptoran dinosaur [17]. Its tail had only 16 caudal vertebrae with the distal ten tightly articulated to form a stiffened rod supporting four unique, ribbon-like, tail feathers.

**Figure 2:**
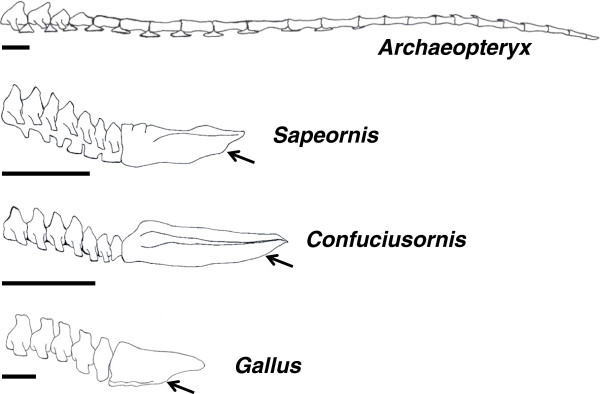
**Comparison of tail skeletons between *Archaeopteryx, Sapeornis, Confuciusornis, *and chicken (*Gallus gallus).*** The *Archaeopteryx* tail was modeled after Gatesy and Dial (1996), as well as the Bavarian, Solnhofen, #11, and Thermopolis specimens. For *Sapeornis,* the tail was reconstructed from specimens IVPP V13276, STM 15-15, and DNHM-D3078. The *Confuciusornis* tail was modeled after Chiappe (2007) and specimens GMV-2131, GMV-2132, and GMV-2133. Pygostyles are indicated by arrows. Scale bars equal 2 cm.

A marked decrease in the number of caudal vertebrae becomes very evident in the bird lineage from the Avialae subgroup of maniraptoran dinosaurs onward (Figure 2). *Archaeopteryx* retained an ancestral caudal vertebral count of between 20 and 23 [18]. The next most basal bird, *Jeholornis*, from the Jiufotang Formation of China and dated at approximately 120 million years old [19], was also long-tailed, and had 22 caudal vertebrae that are nearly identical to those of *Archaeopteryx*[12]. The same formation preserves the next most basal bird, *Sapeornis*[20,21]. This taxon is the first bird to express a shortened tail with only six to seven free caudal vertebrae and an elongated pygostyle. All taxa derived from the last common ancestor of *Sapeornis* and extant birds retain a reduced tail ending in a pygostyle, suggesting that this evolutionary transformation is highly adaptive and necessary for bird physiology. A single specimen, *Zhongornis*, may potentially lie phylogenetically between the long-tailed and short-tailed birds. *Zhongornis* is known from a single specimen representing a juvenile that was either fledged or near fledgling [22]. The juvenile nature of this taxon makes any phylogenetic placement uncertain, but it does have a unique tail morphology that may be either a phylogenetic or ontogenetic signal. Its tail consisted of 13 caudal vertebrae, with the last four partially fused and forming what may be a partial pygostyle.

**Figure 3:**
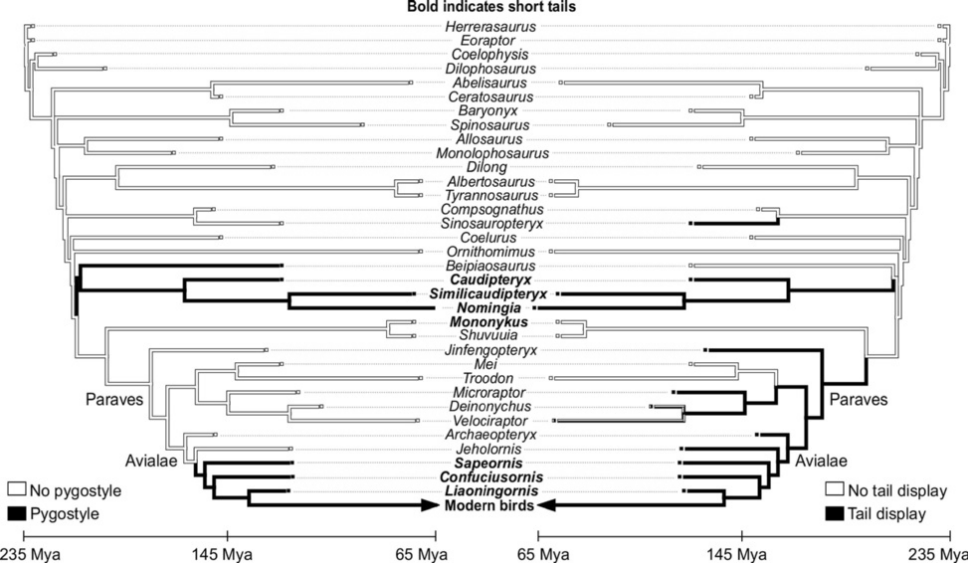
**Evolutionary correlation between the pygostyle, tail length, and possible display behavior.** This mirror tree, constructed in Mesquite [49], shows the correspondence between tail adaptations in theropod dinosaurs, with presence (black) or absence (white) of a pygostyle mapped onto the left tree, and presence (black) or absence (white) of evidence that the tail may have been used in display on the right tree. Note that the tails of species in bold have shortened tails relative to basal theropods.

Despite these variations, there is consensus that short-tailed primitive birds appear in the fossil record relatively suddenly, with fewer caudal vertebrae terminating in a fused distal pygostyle, with abrupt rather than gradual loss of tails [2]. These short-tailed birds, the confuciusornithids, enantiornithines and early ornithurines, had acquired a number of other more modern bird-like traits that differed from their long-tailed primitive bird predecessors. These traits included more extensive synsacral, sternum, and digit fusion (Figure 1), as well as uncinate processes fused to adjacent ribs [23,24]. Osteological modifications were coupled to changes in musculature and behavior. With tail truncation and multiple bone fusions came advances in flight mechanics. Some of those flight advances can be attributed to the pygostyle, partly through its contributions to tail feather control [25]. Because *Jeholornis* had a long tail with a proximal feather fan, there is some debate about whether the pygostyle co-evolved with mobile fan-shaped feather arrays [26]. Whatever their origin, the pygostyle-associated feather fans differed from the frond-type arrays of more primitive long-tailed ancestors [25]. Fan-shaped feather arrays play significant roles in sexual selection in modern birds, and likely played analogous functions in their more primitive short-tailed ancestors (Figure 3) [27].

Truncation of the bird tail was also concurrent with reduction and shortening of the large caudofemoralis muscle (CML). Reduction of this muscle is not exclusive to birds and is evident among all maniraptoran subgroups, as hypothesized from the lack of a clearly distinguishable fourth trochanter, the CML insertion site. More profound CML reductions, however, are predicted in early birds with truncated tails [4]. In theropods and in modern reptiles, the CML originates on the proximal caudal vertebrae, with attachment points on the ventral transverse processes and hemal arches (chevrons). In modern birds, the CML is absent or reduced, and where present, its origination site is on the pygostyle [4]. One exception is the rumpless Araucana chicken; in this case, the CML originates on the pelvis [28]. It is interesting to note that in *Sapeornis*, the most basal short-tailed bird, the caudal vertebrae retained hemal arches [21], but the *Confuciusornis* tail was more derived, and no hemal arches are observed [29] (Figure 2). The presence of hemal arches in *Sapeornis* indicate its CML was more substantial than in *Confuciusornis*, suggesting that formation of the pygostyle alone is not sufficient to cause the degree of CML reduction seen in *Confuciusornis* and in modern birds.

CML modifications, and others within the tail, may have facilitated the abrupt transition to short-tailed birds due to function decoupling. Decoupling of locomotor structures from each other is a hallmark of the origin of birds and powered flight and was most focused in the forelimb and tail [30,31]. The tail of extant birds, for example, functions to provide lift, braking, and turning surfaces for controlled flight [32-36], but is decoupled from the hind limb and has lost its ancestral contributions to terrestrial (as opposed to aerial) locomotion. Therefore, the complex functional repertoire of extant bird tails is achieved by a primary decoupling of the tail from the hind limbs followed by additional flight adaptations within the tail.

### Tail development

#### ***Tail structures***

**Figure 4:**
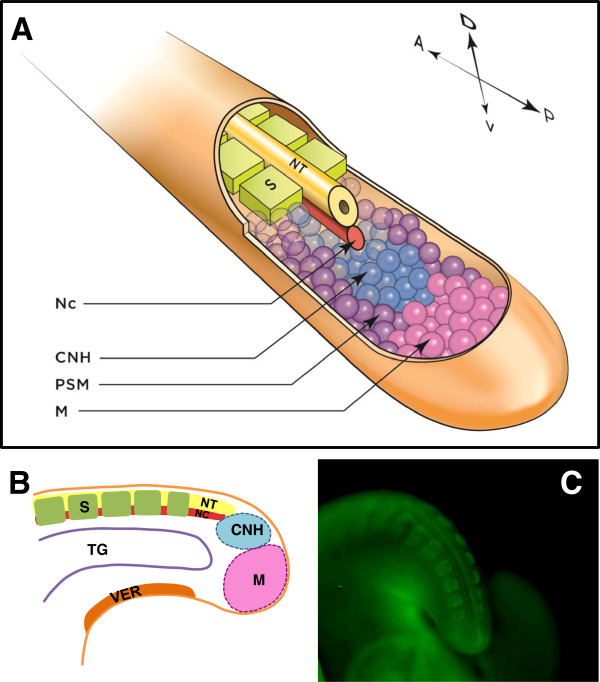
**Structures in the embryonic vertebrate tail. (A)** Three-dimensional (3-D) reconstruction of an extending vertebrate embryo tail. Axial structures include the NT and Nc; lateral to these are the paraxial somites and PSM. Somites are the embryonic precursors to skeletal muscle, ribs, and bony vertebrae; motor and interneurons are derived from the NT; the CNH is the remnant of Hensen's node and contains pluripotent cells; the PSM is the source of cells from which somites arise; and mesenchyme cells (M) at the distal tip of the tail feed into the CNH. Not shown: neural crest and ventral structures. Axis indicates Anterior, A; Posterior, P; Dorsal, D; and Ventral, V. **(B)** Lateral schematic of tail structures. The axial NT and Nc and paraxial somites and PSM lie dorsal to the TG, which in turn is dorsal to the VER. The VER is the remnant of the Hensen's node and a source of growth-promoting signals. Not shown: neural crest and PSM. **(C)** Chick embryo tail stage HH23 stained for somites with FITC-phalloidin. Abbreviations: CNH, chordoneural hinge; M, mesenchyme, Nc, notochord; NT, neural tube; PSM, presomitic mesoderm; S, somite; TG, tailgut; VER, ventral ectodermal ridge.

The mechanisms directing tail growth are similar among vertebrates, and have been evolutionarily conserved since before the dinosaurs. Vertebrate embryo tails are constructed of the same basic elements, arranged in the same basic pattern (Figure 4). Along the dorsal midline lies the neural tube, which gives rise to the brain and spinal cord. Ventral to the floor plate of the neural tube is the notochord, a structure that is the precursor to the nucleus pulposus in spinal discs [37], and is necessary for proper formation of the neural tube and somites [38-40]. Ventral to the notochord in the posterior part of the embryo is the hindgut, the most caudal part of which is known as the tailgut. Somites, discrete paired segments of paraxial mesoderm, flank the neural tube and are the developmental precursors of the axial vertebrae, skeletal muscle and dermis. In addition, neural crest cells, integral to early development, overlay the dorsal neural tube and subsequently migrate ventrally to form the majority of the peripheral nervous system, including the sensory ganglia of the tail [41]. A pool of undifferentiated progenitor mesenchyme cells in the tail bud, the chordoneural hinge (CNH), is the primary source of cells from which tail elongation proceeds [42,43]. Located ventral to the tail tip and adjacent to the forming tailgut is the ventral ectodermal ridge (VER), the remnant of Hensen's node, through which the final gastrulating cells migrate [44-46].

#### ***Axial extension***

**Figure 5:**
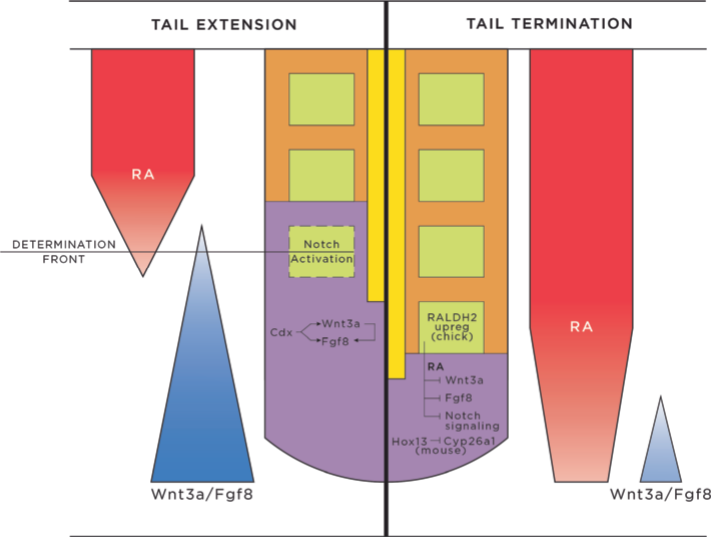
**Tail extension and axial termination signaling schematic.** During tail extension (depicted on left), somitogenesis is actively proceeding, with new somites forming from PSM at the determination front. Activities from Cdx proteins, Wnts, and Fgfs establish a posterior Wnt3a/Fgf8 gradient, which opposes an anterior RA gradient. These opposing gradients allow the creation of the determination front, and activation of the Notch pathway. Cycling expression patterns of Wnt, Fgf, and Notch pathway genes follow a clock wave-front model, promoting somite induction, segmentation and differentiation in successive waves, to add somites sequentially, rostral to caudal, down the vertebrate axis. During tail termination (right), the RA gradient is unopposed, due to progressively decreasing concentrations of Wnts and Fgfs. Contributions from RA (increased in chick via RALDH2), *Hox* genes, decreased concentrations of Cyp26a1 (mouse), Wnts and Fgfs, inhibition of the Notch pathway, apoptosis, and loss of cell division and cell recruitment in the CNH act to terminate the tail. Abbreviations: CNH, chordoneural hinge; RA, retinoic acid.

Early in vertebrate embryo development a body plan is established, whereby somites are added sequentially along the axis. Somitogenesis has been recently reviewed elsewhere [47,48], but in brief, begins with the formation of the presomitic mesoderm (PSM) during gastrulation [49]. Following gastrulation, the region of PSM where somite pairs form is established, at least in part, by the intersection of two opposing extracellular gradients: the Wnt/Fgf gradient and the retinoic acid (RA) gradient. Wnt and Fgf proteins are secreted from the posterior of the embryo, whereas the RA gradient arises from the most recently formed somites. These soluble factors interact at a critical threshold, termed the determination front, where new somite formation is initiated (Figure 5) [50-55]. The addition of somite pairs is controlled by an oscillating 'segmentation clock' signaling cascade, which repeats for each somite pair. The mechanisms guiding the oscillating clock are not completely understood; however, a number of clock participants and their roles have been described [52,56-60]. Among clock genes with time-dependent oscillating expression patterns are members of the Wnt, Fgf, and Notch pathways. The cooperative action of the molecular pathways functions to synchronize the oscillation of the clock, such that a wave front of clock-gene expression moves anterior to posterior along the embryonic axis. Negative feedback regulation of clock genes by their targets within activated cells as well as RNA instability are mechanisms employed to generate oscillating gene expression [61-63]. The boundaries of newly formed somites are established by positional expression of Notch pathway genes; these genes also establish the anterior/posterior axis of each somite [64-68]. As somites are sequentially added, ingression through the primitive streak and cell division in the PSM and CNH feeds into and maintains the PSM for continued somitogenesis [42]. Krol and colleagues conducted a particularly interesting study comparing the transcriptomes of mouse, chicken and zebrafish during one somite extension. They discovered that despite a high level of conservation of the major pathways and events of somitogenesis, the genes that show oscillating expression can differ. Only two Notch pathway proteins, Her1 and Her5, were shown to oscillate in all three vertebrates, but all other identified oscillating proteins, primarily members of the Fgf, Notch, and Wnt cascades, were specific to each vertebrate. This suggests an unexpected evolutionary plasticity in a critical developmental process. Specifically, members of the Fgf, Notch, and Wnt pathways were likely targets of evolution in axial extension [69].

#### ***Regional specification***

The regional identity of the somites, that is, cervical, thoracic, lumbar, sacral or caudal, is determined by *Hox* gene expression [70]. The *Hox* genes were first discovered in *Drosophila*, where *Hox* gene mutations changed the positional identity of segments along the *Drosophila* body axis [71]. *Drosophila* and other non-vertebrates have up to 14 genes contained within one *Hox* cluster. Due to tandem genomic duplications, vertebrate *Hox* genes usually appear in four paralogous DNA clusters, A through D. *Hox* genes within those clusters, numbered 1 through 13, are collinearly expressed along the body axis sequentially, with *Hox1* most rostral and *Hox13* most caudal [72-77]. In any given vertebrate or non-vertebrate organism, not all 13 or 14 *Hox* genes within each paralogous cluster are present [78]. Teleost fish sustained an additional genome duplication, and therefore, possess another set of *Hox* clusters. While four more *Hox* clusters would be expected, three have been identified, bringing the total number of clusters in teleosts to seven [79]. In vertebrates, *Hox* genes perform analogous body patterning functions to *Drosophila* and are most evident in defining the rostral to caudal identities of vertebrae. Most *Hox* genes are thought to specify regional axial identity by initially conferring anteroposterior patterning during gastrulation [80], followed by fine-tuning within maturing mesoderm and neuroectoderm (reviewed in [81]). Mutations in *Hox* genes typically cause homeotic transformation, in which vertebrae take on characteristics that are more anterior or posterior to their position. Concurrent disruptions in all three mouse *Hox10* genes, for example, cause the lumbar vertebrae to transform into thoracic-like vertebrae with ribs [82]. Conversely, loss-of-function of the more posteriorly expressed three *Hox11* genes in mice results in a failure to form sacral vertebrae, being replaced by vertebrae with lumbar morphology. While these mutations generally preserve the overall number of vertebral elements, some *Hox* gene disruptions can increase or (more commonly) decrease total vertebrae numbers (reviewed in [78]).

There are additional factors that contribute to regional specification of the tail. *Gdf11*, for example, which encodes a Bmp (Bone morphogenetic protein)-related growth factor, acts to establish the trunk-to-tail transition in vertebrates [83]. Also involved in caudal axial patterning and posterior expansion are the *Cdx* genes, which are expressed primarily in the posterior end of the vertebrate embryo. *Cdx* and *Hox* genes are related, and are thought to have derived from the same ProtoHox cluster [84]; both have been highly conserved throughout evolution and play roles in body patterning from cnidarians to higher vertebrates. Three separate *Cdx* genes are generally expressed in vertebrates, and if two of these are knocked out in the mouse (*Cdx2* and *Cd*x*4*), axis extension is prevented and some trunk and tail structures do not form [85]. Control of *Cdx* and *Hox* genes is mediated through the Fgf, Notch, RA and Wnt signaling pathways, underlining the crucial contributions of these pathways to extending and shaping the spinal column [78,86,87].

#### ***Caudal positional identity***

The correlations between *Hox* gene expression pattern and vertebral specification are not simple, and functional redundancies are prevalent. In both chick and mouse, the anterior expression boundary of *Hoxd12* marks the transition between sacral and caudal vertebrae [88]. *Hoxd12* contributes to caudal specification, but its disruption in the mouse causes limb abnormalities [89]. In the mouse, loss-of-function studies have shown that caudal vertebrae are also specified by *Hoxb13*[90]. Adding further complexity to the system, *Hoxb8* in the mouse is expressed in mesoderm in the tail (and also more anteriorly). *Hoxb8* knockout does not alter caudal vertebrae identity [91], but it can rescue tail vertebrae in *Cdx2/4* mouse mutants [81], indicating that it may contribute to caudal somite extension. Further genetic analyses will help to determine whether the functions of these *Hox* genes in the tail are universal in vertebrates.

#### ***Tail termination***

Whether a tail is long, like that of a mouse, or short, as in a chicken, a number of converging developmental events ensure that the tail stops elongating at a characteristic point in each species and does not grow indefinitely. At the termination of axial extension, secondary body formation is completed, with somites, neural tube, and notochord development concluded. At this point, the pool of progenitor mesenchyme cells within the CNH is depleted by a combination of events: epiblast migration through the VER is complete, the non-proliferating mesenchyme that feeds into the CNH population is also depleted and the neuroblast cells originating in the CNH population have exited the cell cycle. Since most of these structures and cell populations are required for axial extension, the loss of nearly any one of them will halt progression of the tail. The vertebrate embryo hedges its bets, however, and has distinct concurrent mechanisms for terminating some if not all of these elements (Figure 5).

**Figure 6:**
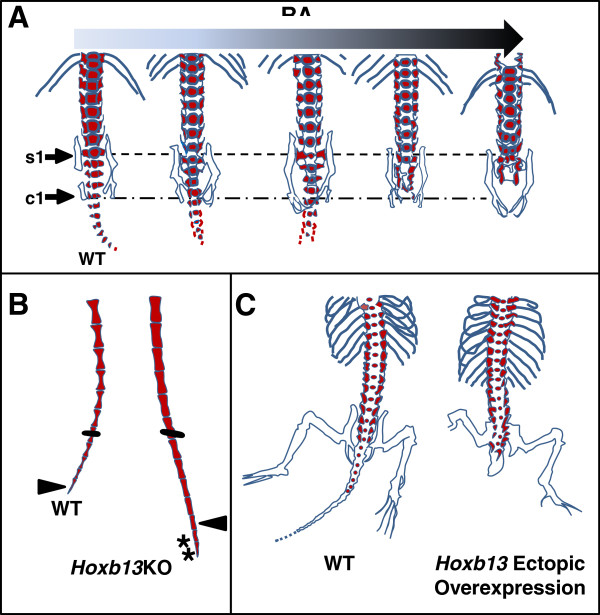
**Experimental manipulations affecting the length of the vertebrate tail. (A)** Increasing RA exposure in mouse embryos leads to progressive loss of caudal and sacral vertebrae. s1 indicates first sacral vertebrae and c1 indicates first caudal vertebrae. Data adapted from Shum *et al*. 1999 [93]. **(B)***Hoxb13* knockout (*Hoxb13*KO) in the mouse increases caudal vertebrae number by 2 and causes more barrel-shaped as opposed to hourglass-shaped vertebrae. Bars indicate experimental marking of equivalent numbered vertebrae; arrowheads indicate caudal vertebra #30 in both wildtype (WT) and *Hoxb13*KO; asterisks indicate two additional caudal vertebrae. Data adapted from Economides *et al*. 2003 [90]. **(C)** Precocious ectopic overexpression of *Hoxb13* in the mouse causes prematurely truncated tails. Data adapted from Young *et al*. 2009 [81]. RA, retinoic acid.

When somitogenesis is nearing completion, the rate of cell addition to and somite separation from the PSM begin to slow down. In the chick, the first 45 pairs of somites take 90 minutes each to form; thereafter, the segmentation clock slows such that the final 5 to 8 caudal somites (predecessors of the pygostyle) take 150 minutes to form [42]. One likely candidate for this deceleration is WNT3a. While somitogenesis is robustly proceeding, up to HH (Hamburger Hamilton [92]) stage 21, WNT3a is highly expressed in the tail mesenchyme, but as somite addition and the need for PSM nears its close, WNT3a is gradually downregulated between HH22 and HH25 [42]. RALDH2, an enzyme involved in the synthesis of RA from vitamin A, is correspondingly upregulated in the tail mesenchyme in the chick and is thought to be responsible for WNT3a and FGF8 downregulation. This creates an imbalance of signaling factors, and thereby promotes the effects of RA. Exposing mouse embryos to increasing levels of RA induces more severe axial truncation (Figure 6A) [93]. In addition to contributing to the loss of the growth factor Fgf8, RA causes differentiation of somitic cells and concomitant reduction in cell division; without Wnt3a, these effects act as barriers to further somite addition. In the mouse, RA is also promoted by downregulation of Cyp26a1 [42], an enzyme that normally metabolizes RA [94]. When Cyp26a1 is downregulated, RA concentration effectively increases, further inhibiting tail growth [95,96]. Interestingly, a critical level of RA signaling is required, as either augmenting or decreasing the amount of RA causes premature termination of somitogenesis [93,97,98]. In this finely tuned system, RA is required for maturation of recently made somites before the next pair of somites can form, but prolonged exposure to RA prevents further somite addition. Wnt3a expression also affects somitogenesis via its cross-talk with the Notch pathway. Specifically, Wnt3a and Notch pathway genes regulate each others' expression levels and patterns [57,99-103], and Notch pathway genes are intrinsically tied to the segmentation clock and somite boundary formation [47,48,104]. As an indication of the coordination between these various pathways, loss of Fgf4 and Fgf8 in the mouse tail PSM results in loss of Wnt3a, downregulation of Notch signaling, and inhibition of Cyp26a1 [99], all of which act together to prematurely truncate the tail. Since Wnt3a also influences the expression of Fgf8 [103], downregulation of Wnt3a at the end of somitogenesis inhibits tail growth by influencing both RA effects as well as through inhibition of Notch-directed somite formation and maturation.

Changes in the VER occur as tail growth comes to an end, terminating notochord elongation, inhibiting somitogenesis [105], and also terminating caudal gastrulation [106]. The thickened ventral epithelium that characterizes the VER reaches its peak at approximately the 45-somite stage in the chick; subsequently, the VER gradually diminishes until it disappears altogether [105]. The dissipation of the VER is concomitant with loss of its signaling. Before the VER declines, it expresses several secreted proteins, including Sonic Hedgehog (Shh) [107], Fgf17, Bmp2, and Wnt5a [105,108], and functions to control the expression of Noggin in overlying ventral mesoderm [106]. Noggin, in turn, is a powerful modulator of Bmps, and hence plays significant roles in Shh/Bmp signaling cascades. As the VER recedes, it can no longer maintain its signaling, and Noggin expression in ventral mesoderm is downregulated. When the VER is ablated, it prevents the ingression of epiblast cells that are needed for feeding cells into the non-proliferating mesenchyme of the tail and thus, the CNH. The neural tube can temporarily extend without the presence of the notochord (albeit with ventral patterning abnormalities) [38,39], but somite formation and patterning are disrupted [40], and the tail prematurely truncates. Thus, the indirect effects of VER disruption affect the tail progenitor population and lead to termination of tail elongation.

Ablation studies, in which different axial structures are removed in a living embryo, have shed light on the interdependence of a number of these structures for axial extension. As mentioned above, VER or notochord ablation results in the failure to form the complete secondary neural tube and caudal somites, and leads to premature axial truncation. In studies where the neural tube was ablated, the dermamyotome region of differentiated somites was absent, likely due to disruptions in Bmp and Wnt signaling from both the dorsal neural tube and overlying dorsal ectoderm [109]. Removal of the CNH population causes subsequent loss of neural tube, notochord, somite formation inhibition and shortened tails [39,43]. If the somites themselves are removed, neural crest cell delamination and subsequent migration come to a halt; an imbalance between Noggin and Bmp4 is thought to be responsible [110]. Reminiscent of the balance between Wnt3a, Fgf8 and RA, there is also a balance between Noggin, Shh and Bmps between different structures in the tail. The opposing functions of these proteins help to pattern the neural tube [111] and are also involved in somite segmentation and differentiation [112]. Disruption of this balance likely plays multiple roles in terminating elongation of the tail.

Applying information from all of these ablation studies to the normal process of tail termination has helped us to understand why the disruption of one structure leads to the termination of others, and helps to explain why peripheral and motor nerves are missing at the end of the tail. Just as Noggin is upregulated with somite ablation, it is also upregulated in the dorsal neural tube at the end of the naturally waning tail. This enhanced expression is thought to inhibit neurogenic neural crest derivatives, causing loss of peripheral nerves [113]. Lack of motor neurons just anterior to the end of the tail is likely due to termination of the neural tube (from which motor neurons derive [114]), the loss of Shh signaling from the notochord and neural tube [115], which is required to pattern the motor neurons in the ventral neural tube, and/or the presence or absence of an as-yet unidentified diffusible signal intrinsic to the tail [116].

Another process involved in tail cessation is apoptosis, or programmed cell death. Apoptosis is a mechanism employed by embryos to help sculpt morphological features and cull extraneous cells, and is often considered to be a default pathway for those cells that find themselves within inappropriate environments. During axial extension, apoptosis is kept at bay at least in part by survival promoting signals through the Shh/Noggin/Bmp cascade [39,117], but this cascade is largely disrupted as tail growth slows. At these later stages, there is considerable cell death in the tail bud [118], which depletes the available pool needed for somite production. Signaling from Wnt3a and Fgfs is necessary for maintaining the pool of undifferentiated PSM cells which give rise to somites [55,119], and downregulation of these pathways in the waning tail contributes to apoptosis. In higher vertebrates, this severe reduction in PSM, however, is caused only in part by apoptosis. Due to RA effects, there is reduced cell division [42,120], and neuroblast cells exit the cell cycle, further depleting the progenitor population. However, PSM has not been completely eradicated before the segmentation oscillator comes to a halt, and the remaining PSM becomes unresponsive to signals that promote axial extension [42]. The concept that apoptosis plays a contributing but not solitary role in tail cessation is further substantiated by the fact that significant mesenchyme cell death occurs even while the tail is robustly extending [121], and apoptosis has been inhibited in different vertebrates, but longer tails have not been documented [122].

Finally, the prescribed species-specific number of somites that are formed is controlled by *Hox* genes [123,124], and the most caudally-expressed *Hox* genes act to terminate tail elongation. Precocious over-expression of *Hox13* paralogs at the posterior end of mouse [81] and chick [125] embryos leads to prematurely truncated tails with loss of caudal vertebrae (Figure 6C). Conversely, targeted knockout of *Hoxb13* in the mouse leads to expansion of tail structures, including neural tube, PSM, and two extra caudal vertebrae (Figure 6B). A reduction in the level of apoptosis in PSM was also observed in these knockout mice, which ties *Hox13* genes to depletion of the mesenchyme cells needed for somitogenesis. In this same study, *Hoxb13* was also shown to inhibit neuronal proliferation, which, combined with the normal loss of caudal neural crest-derived neurogenic cells, doubly ensures the lack of spinal ganglia at the end of the tail [90]. Another potential mechanism *Hox13* genes employ to terminate the tail is to intersect the Wnt/Fgf/RA gradient by downregulating Cyp26a1 [81], providing yet another example of the level of involvement of this gradient on tail cessation.

To summarize, perturbations in virtually any of the tail elongation processes described above lead to termination of extension, and numerous perturbations are built into the axial extension system to ensure proper tail length. Imbalances in the Wnt/Fgf/RA and Noggin/Shh/Bmp gradients are largely responsible for stopping tail growth. Once disrupted, the signaling cascades generated from the gradients no longer properly coordinate with other cascades such as Notch, thereby disabling the elongation machinery. *Hox13* paralog genes further inhibit tail elongation, likely through their interactions with the regulatory factors that control these gradients [81]. Finally, increased apoptosis at the termination of somitogenesis removes all remaining progenitor cells. All of these coordinating pathways are orchestrated through the different tail structures, and signaling between the structures maintains their inter-dependence so that if even one structure fails, the rest eventually follow suit. Species-specific differences in the way the orthologous pathways are modulated likely account for the varying tail lengths observed among vertebrates [123,124].

#### ***Skeletal development of the bird synsacrum and tail***

**Figure 7:**
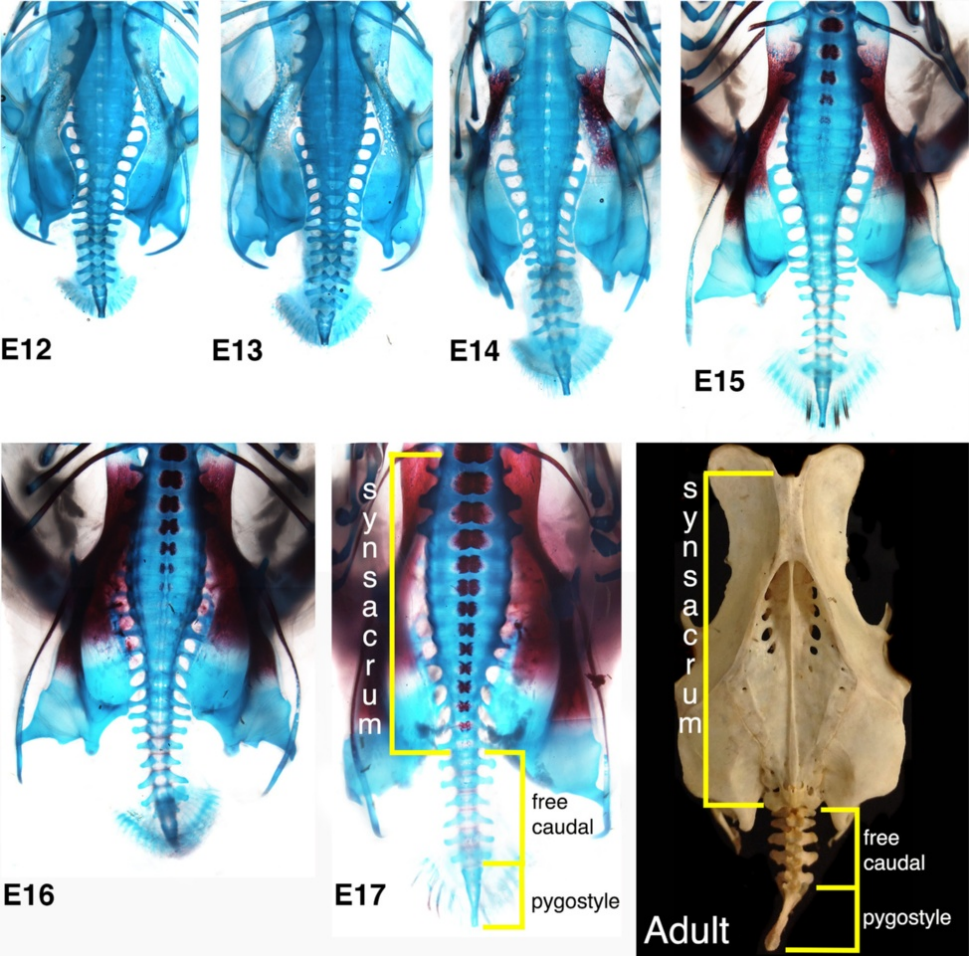
**Embryonic events during the termination of the chick embryo tail.** Embryonic day, E12 to E17 chondrified skeletons (blue) of chick embryos, with ossified cells (red) detectable from E14 to E17. Compare the E17 chondrified skeleton and the adult skeleton showing the fused synsacrum and bony plate in the latter; the 5 free caudal vertebrae and the pygostyle already patterned during somitogenesis.

Following the formation of somites that will contribute to the synsacrum, an axial structure with 14 fused thoracic (1), lumbar (6), sacral (2) and sacro-caudal (5) vertebrae (Figure 7) [126] (in chick, there is no evidence that caudal vertebrae are incorporated into the synsacrum [88]), the chick tail achieves its maximum number of somites by E5. Although the synsacral somites form as separate blocks of tissue, the chondrified synsacral cartilages fuse together to form a continuous structure, devoid of intervertebral discs. Distinct ossification centers for each of the vertebrae are retained, with the onset of ossification observed in a rostral to caudal sequence from E15 onward (Figure 7). In addition to the centrum of the vertebrae, the free sternal ribs have ossification centers. The lumbar vertebrae that follow have transverse processes, but these do not have independent ossification centers, rather ossifying from the pedicle situated between the centrum proximally and the transverse processes distally. The ventral processes abut and become fused to the ilium. Notably, the transverse dorsal processes and dorsal ligament uniting the lumbar vertebrae ossifies postnatally forming a continuous plate of bone, or sacral shield (Figure 7, adult). This is a common feature of birds from neornithines to modern birds, helping to strengthen the fused synsacrum [1]. The rigid synsacrum and ilium fuse to form an immovable structure with osteoblasts visible in the ilium from E14. The transverse and ventral processes of the two sacral vertebrae abut and fuse to the medial posterior curve of the ilium. These processes are sometimes referred to as sacral ribs, having their own ossification centers, similar to sternal ribs [127]. Beyond the synsacrum, the free caudal vertebrae develop ossification centers at E18, and finally, by E19 the fused cartilaginous elements of the pygostyle follow suit (not shown). Ossification of the axial vertebrae and pelvic girdle is complete by hatching [126]. Extending beyond the synsacrum, the mature tail in the chick consists of 5 to 6 free caudal vertebrae (there are 5 to 8 free caudal vertebrae among birds in general) and the pygostyle (a fusion of the final 5 to 6 somites).

### Mutations that cause tail truncation

Relating the developmental events of axial extension and termination back to the process of evolution, one needs to consider birds as organisms that sustained one or more mutations that converted long theropod tails to short avian tails terminating in a fused, distal pygostyle. Considering the many redundancies in the process of tail cessation, it follows that just one mutation could have truncated the posterior axis. Alternatively, the short, fused tails of early birds could have been the result of a suite of mutations that occurred over a longer period of time, and the fossil record is incomplete. Complicating the genetics behind the transition to short-tailed birds is the nature of the mutations that could have occurred. Mutations can occur within gene coding sequence, in *cis* regulatory regions (CREs) outside coding sequence that control gene expression, by DNA deletion, or by gene duplication [128-130]. The prevailing theory is that most phenotypic changes in evolution are due to changes in CREs [128]. Alterations in the regulation of gene expression would allow for fewer pleiotropic and potentially deleterious effects of critical genes, by affecting some but not all expression patterns. Despite the potentially higher chance that changes in CREs were responsible for short fused tails, any of the other above-mentioned mechanisms were possible. It remains to be asked, given the lack of dinosaur DNA, how can we parcel out those mutations that affect morphological changes in the tail and may have converted theropod tails to bird tails?

One way to study the ancestral ties between organisms is to proceed with an evolutionary developmental biology or 'evo-devo' approach. This approach is particularly appealing when studying theropod-to-bird evolution, because despite the lack of dinosaur DNA, we can still examine gene pathways that potentially generated dinosaur traits. In terms of tail morphology, the gene pathways that are involved in tail elongation and termination in different organisms can be studied side-by-side, and modulations of those pathways that generate long versus short tails can be compared. In considering the many pathways involved in tail elongation and cessation, how do we narrow down the list of candidate genes that may have been modulated by mutation? For this particular study, we looked to the mouse, the vertebrate organism with the greatest accumulated data on mutations. Most mouse mutational data has been generated by targeted gene disruption, which causes phenotypes that are likely more extreme than mutations that would occur in, say, CREs. Despite the preponderance of targeted transgenesis, substantial mutational information has also been contributed by chemical, radiological, or transposon induction of random mutations, as well as by studies of spontaneous mutations. However the mutations occurred, the mouse is a reasonable place to begin the examination of those genes whose modulation affects tail morphology.

#### ***Morphological analysis of mouse mutants***

A list of mouse tail mutants was generated from the MGI Jackson Laboratories database [131] and the literature [see Additional files 1 and 2]. From this list, a number of interesting and surprising correlations surfaced. Immediately obvious was the observation that of the 159 mutants with affected tails, only two, the *Hoxb13* (Figure 6B) and *Slx4* knockout mice, have increased numbers of caudal vertebrae, and these mutations cause only modest increases. Indeed, the tail suffers from a particular developmental precariousness, as seen in the preponderance of mutations causing short tails, suggesting that tail growth is relatively easily disrupted. While this remains to be studied across vertebrates, in this particular case, one could propose the argument that the early decoupling of the tail from hind limb locomotion in maniraptoran theropods may have facilitated tail reduction through a process of relaxed purifying selection. Relaxed purifying selection has been demonstrated to promote phenotype plasticity [132], and thus, may also facilitate rapid evolutionary change. The distal portion of the tail, once completely decoupled from hind limb function, may have been relatively free to accumulate mutations without deleterious effects and thereby facilitate the evolution of novel morphologies, namely a radically shortened tail and pygostyle.

To correlate the mouse mutants with specific skeletal differences observed between theropods, primitive birds and modern birds, several parameters were taken into consideration. When modern bird tails are compared with those of their more primitive bird or non-avian theropod ancestors, there are three primary differences: reduction in the number of caudal vertebrae, shortening of the caudal vertebral bodies, and fusion of the most distal caudal vertebrae into the pygostyle [25]. Bone fusion is indeed highly evident in the modern bird skeleton. Fusions are observed not just in the pygostyle, but also in the synsacrum and in the dorsal vertebrae anterior to the synsacrum, between the ribs as cross bridges called uncinate processes, and in the distal limbs. Between 150 and 120 million years ago, long before the Cretaceous Tertiary Extinction, these modern bird traits were evolving in primitive birds and other maniraptoran dinosaurs, and a number of variations of these traits have been observed in fossil specimens from this timeframe [2]. From the accumulated list of short-tailed mouse mutants, it is evident that most mutations affected more than just the tail, and a whole host of other pleiotropic defects were also observed, including, among others, more anterior bone fusions [see Additional file 1]. The question then becomes, are there any morphological traits that co-segregate with reduced numbers of caudal vertebrae for single mutations, and do any of these traits co-segregate in the fossil record? Fusions between various vertebral surfaces are observed in the bird skeleton, so different types of fusions were considered. Among the mouse mutants with decreased numbers of caudal vertebrae (n = 105), it was interesting to note that 34% (36/105) also displayed vertebral fusions (including fusions of neural arches, articular surfaces/zygopophyses, transverse processes, spinous processes, or vertebral bodies). Of the 36 with vertebral fusions, 53% had fused ribs. Of all mutants with decreased numbers of caudal vertebrae, only three also had digit fusion, which does not constitute a significant degree of co-segregation but indicates that digit fusion is also possible with truncated tail mutations.

Because modern and primitive short-tailed birds exhibit both truncated tails and fused vertebrae [1,2], we asked the opposing question: If a mouse mutation transpired which caused caudal vertebral body fusion (the predominant type of vertebral fusion observed in the pygostyle of modern and short-tailed primitive birds), what was the chance that caudal vertebrae number was also decreased? Seventeen of 23 caudal vertebral body fusion mutants, or 74%, also had truncated tails (Table 1). A high percentage of caudal vertebral body fusion mutants (48%) also displayed fused ribs. Thus, in the mouse, if a caudal vertebral body fusion event occurred, there was a nearly even chance the mouse also had fused ribs and a significantly better than even chance that it also had a truncated tail (for a complete list of mouse posterior vertebral fusion mutants and additional information on the caudal vertebral fusion mutants, see Additional files 2 and 3). Since there is a fairly high correlation of vertebral fusion, rib fusion, and truncated tails with mouse mutants, we next asked whether these traits also co-segregate in the fossil record in the transition from non-avian maniraptorans and primitive long-tailed birds to short-tailed birds.

**Table 1:** Caudal vertebral body fusion mouse mutants

**Mutant**	**Affected vertebrae**^**a**^					**cdl v #**^**b**^	**Fused ribs**	**Fused digits**	**Structure affected**				**Relevant pathways**
	**C**	**T**	**L**	**S**	**cdl**				**So**	**NT**	**NC**	**VER**	
*Ankrd13a*					✓								Nd
*Cenpj*					✓			✓					Nd
*CREB*	✓	✓	✓		✓		✓		✓				Notch/Wnt
*Dkk1* doubleridge/null			✓	✓	✓			✓	✓				Wnt
*Dll3*	✓	✓	✓	✓	✓	++	✓		✓				Notch/Wnt
*f* flexed tail; Sfxn1 mutation			✓	✓	✓	+/-	✓			✓	✓		Bmp/Shh
*Fgf3*					✓	++			✓	✓			Notch/Wnt
*Hes7*	✓	✓	✓	✓	✓	++	✓		✓				Notch/Wnt
*Ikkα*	✓			✓	✓	+		✓					Notch; FGF
*Jsr* jumbled spine and ribs		✓			✓	++	✓		✓				Notch/Wnt
*Knk* kinked tail					✓	+					✓		Notch; Wnt
*Lrp6* crooked tail					✓	++	✓		✓	✓			Notch/Wnt
*mea* meander tail		✓	✓	✓*	✓	++							Wnt
*Meox1/Meox2*					✓	++	✓		✓				Retinoic Acid
*Noto*			✓	✓	✓	++			✓	✓	✓		Wnt
*Nrarp*		✓	✓	✓	✓	+	✓		✓				Notch/Wnt
*Ppp5c*					✓								Wnt
*Ror2*					✓	+	✓	✓	✓				Notch/Wnt; Wnt
*Rpl38*	✓	✓			✓	++	✓			✓			Hox
*Rps7*				✓*	✓	+							nd
*Sulf1/Sulf2*		✓	✓	✓	✓				✓				Bmp/Shh
*Vangl2*		✓	✓	✓	✓	++	✓			✓			Wnt
*Wnt5a*				✓	✓	+++			✓	✓		✓	Wnt

Selected phenotypes and relevant genetic pathways for each mutant are indicated**. **^a^Asterisks indicate fusion other than vertebral body fusion. ^b^For the decreased number of caudal vertebrae (Cdl vertebrae #) column, **+**: less than half of the tail is absent; ++: half or more of the tail is absent; and +++: tail is severely truncated. Check marks indicate the presence of a particular trait. For a more comprehensive list of posterior vertebral body fusion mouse mutants (those which have fusions posterior to the cervical vertebrae, as seen in modern birds), and for more information on the caudal vertebral body mutants, see Additional files 2 and 3. *Abbreviations:* C, cervical; cdl, caudal; L, lumbar; NT, neural tube; NC, notochord; nd, not determined; S, sacral; So, somite; v, vertebrae; VER, ventral ectodermal ridge.

Correlations between certain maniraptoran traits have been noted before, especially the co-incidence of short tails and the presence of a pygostyle [133]. The co-segregation of short tails and a pygostyle applies to a wide range of feathered non-avian maniraptorans and primitive birds, lending credence to the possibility that the traits are pleiotropic and linked by a single mutational event, irrespective of adaptive advantage. This is not to suggest that the same mutation occurred in these different groups; the number of different mouse mutants with these traits indicates that a number of different genes were likely causative. It should be noted that not all dinosaurs with a pygostyle had short tails (for example, *Beipiaosaurus*[134]). These long-tailed dinosaurs with a pygostyle, however, were in the minority, just as are the mouse mutants with fused distal vertebrae and unaffected tail length.

In the mouse mutants analyzed, vertebral fusions were correlated not just with short tails, but also with fused ribs. Rib fusions are likely observed with these mutations because somitogenesis is the most common developmental event affected, and vertebral ribs arise from somites [135-138]. If rib fusions were coincident with a vertebral fusion mutation, they could have altered the axial skeleton in a few different ways. Very proximal rib fusions could have helped establish the wide dimensions of the ilium (synsacrum) by increasing bone mass at the point ribs attach to the axial skeleton. Very distal rib fusions could also have increased the breadth of the sternum, as seen in the *Gnai3* mouse mutant [139]. Branching of ribs is occasionally observed in some mutants, such as *Tbx6*[140,141] [see Additional file 3], which could have been the mechanism behind the formation of uncinate processes. This possibility seems less likely, however, considering that quite a number of non-avian theropods exhibited uncinate processes with long tails and no pygostyle, such as *Oviraptor philoceratops*, *Velociraptor mongoliensis*, and *Diononychus antirrhopus*. Also, *Sapeornis* had a short tail with a rod-like pygostyle and no uncinate processes [21]. Even if uncinate processes had already evolved through separate means, however, we hypothesize their fusion to adjacent ribs (seen only in Aves [23]) could have been facilitated through a vertebral fusion mutation.

#### ***Genetic analysis of mouse mutants***

Investigation into the genetic pathways that are modulated by these caudal vertebral body mutations in the mouse also proved insightful (Table 1 and [see Additional file 3]). Of the 20 (of 23) mutations whose affected pathways were previously studied, 10 involve Notch or Notch/Wnt signaling. The remaining mutations have the following pathway associations: seven involve Wnt signaling (possibly independent of Notch), two are associated with BMP/Shh cascades, and one each is involved in Hox or RA signaling. The genes that were mutated appear to have developmental roles that would be expected, which include functions in somitogenesis (most prevalent), neural tube and notochord biogenesis and patterning, mesoderm establishment and maintenance, neurogenesis, angiogenesis, and VER signaling. Of those associated with the Notch pathway, the majority (6/10) were involved in somite segmentation or differentiation; these include *CREB*, *Dll3*, *Fgf3*, *Hes7*, *Lrp6*, and *Nrarp*. It is intriguing to note that in the chick, the Notch pathway members *Lnfg*, *Nrarp*, and *Meso* (the chick homolog of *Mesp2*), are all downregulated as somitogenesis slows [42], at an equivalent point at which the mouse tail would still be actively extending. Interestingly, several mutations among members of this particular pathway, including *Dll3*, *Hes7*, *Lnfg*, *Lrp6*[142], *Mesp2*, and *Tbx6*, are reasonably well tolerated and cause spondylocostal dystosis (SCD) disease in humans [3]. Individuals suffering from this disease display fused ribs and vertebrae with unaffected reproductive capacity, as in the mouse mutants.

#### ***Experimental manipulations and one spontaneous mutation that affect chick tail morphology***

As in other vertebrate species, the chick tail is often neglected as a focus of research. There are, however, additional studies that deserve mention here apart from the RA and *Hox13a* manipulations already cited. To date, targeted transgenesis in the bird is largely unreported, and even transgenic overexpression has been restricted to a small handful of genes. Genes or proteins can be modulated in other ways, however, and the chick embryo is amenable to studies such as microinjection, electroporation of DNA or RNA, viral transfection, and insertion of matrices soaked with diffusible proteins or other factors. The specific morphological changes upon ectopically applied RA in the tailbud are particularly interesting. In addition to posterior truncation, stretches of accessory neural tube and notochord occur when RA is injected into the tailbud, indicative of enhanced neural differentiation [143]. Premature neural differentiation is evident in a number of the mouse mutants with truncated tails [see Additional file 1] and is also evident in the rumpless Araucana chicken [144]. To our knowledge, no manipulations in the bird have resulted in longer tails with increased numbers of caudal somites/vertebrae. One study attempted to extend neural crest in chick embryo tails by inhibiting Noggin [113]. While certain neural crest markers were indeed upregulated at the end of the tail, additional somites were not added and tail length remained unchanged. Just as in the mouse, manipulations in the tail are far more likely to reduce or otherwise fail to alter length as opposed to increase tail length.

**Figure 8:**
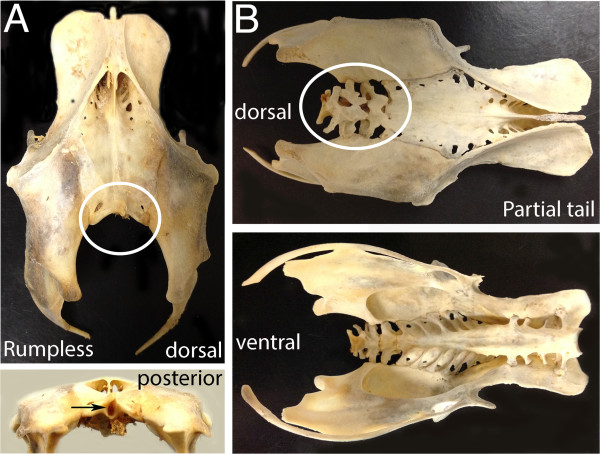
**The Araucana rumpless chicken mutant.** Adult skeletons showing the pelvis, synsacrum and caudal vertebrae. **(A)** In the homozygous mutant no caudal vertebrae develop (circle). The synsacrum develops normally and the bony plate over the fused vertebrae forms as expected. There is a hole in the final vertebra leaving the neural tube exposed. **(B)** In the heterozygote, 2 to 4 fused elements form beyond the synsacrum, in place of the free caudal vertebrae.

The only known spontaneous mutation that truncates the avian axial skeleton, namely in the rumpless Araucana chicken (Figure 8A), was identified as a gain-of-function mutation of the proneural *(Iroquois*) *Irx1* and *Irx2* genes [145]. Iroquois genes are tied to Notch, Wnt, and Bmp/Shh signaling [146-148], and in addition to their proneural role, they establish tissue borders during development. Interestingly, heterozygotes of the rumpless locus retain 2 to 4 caudal vertebrae, and these are irregularly fused (Figure 8B) [149,150], adding this mutation to those that cause both short tails and fused vertebrae. The most caudal somites are never generated and the pygostyle, therefore, never forms. While there is no equivalent gain-of-function mouse mutant, loss-of-function mutations in either *Irx1* or *Irx2* in the mouse do not cause posterior truncation or fused vertebrae, emphasizing an important caveat with this study that mutation of the same genes can be manifested differently depending on the nature of the mutation.

It was previously estimated that among a variety of normal-tailed chicken breeds, a tailless phenotype was consistently observed in approximately one out of every thousand chicks hatched, making further tail truncation a relatively common chicken mutant phenotype [151]. Well-tolerated, relatively common tail truncating mutations (especially if dominant and germ-line) that conferred certain advantages would theoretically promulgate the evolutionary transition from long- to short-tailed birds.

#### ***Additional considerations***

If a single mutation occurred that shortened the tail and fused the distal caudal vertebrae in short-tailed primitive birds, it would seem logical that occasionally, that gene modulation would spontaneously reverse resulting in more recent long-tailed birds. Several lines of evidence contradict this possibility, however. First, while not impossible, direct reversals of mutations are extremely unlikely. Indirect mutations that would re-activate a particular pathway are possible but also unlikely, considering the mutation(s) would have to reintroduce the fine balance of factors required for axial extension without being detrimental. Second, Dollo's law states that accumulation of mutations over time, especially for those genes whose functions are no longer selected for, can prohibit certain morphological traits from resurfacing [152]. Birds with fully formed teeth, for example, have never been observed in modern times because the genes for enamel, no longer selected for, were inactivated in the bird genome [153,154]. Third, since long tails hinder flight, and flight mechanics evolved primarily with short tails [36], reintroduction of long tails would have likely impeded survival. Lastly, traits that influence sexual selection cannot be underestimated. The appearance of the pygostyle, possibly with the fan-shaped array of mobile tail feathers, may have indelibly affected mate choice among birds, which would have ensured the persistence of the pygostyle phenotype.

The ramifications to a long-tailed bird that suddenly lost the bulk of its tail also need to be considered. An anterior shift of its center of mass would likely have impacted flight and terrestrial locomotion. Hutchinson, however, postulated that the reduction of the caudofemoralis muscle in primitive short-tailed birds would not have impeded them from being very capable runners [155]. Also, an anterior shift of the center of mass, observed in primitive and modern birds, is modeled to facilitate flight [30].

## Conclusions

We have no way of knowing at this point, of course, what or how many mutations occurred in early bird evolution, or even which was the true basal ancestor that sustained the initial mutations. Despite the many caveats with this approach, however, our analyses of genetic and fossil evidence suggest the possibility that a single mutation could have occurred in a paravian dinosaur, which both truncated its tail and fused its distal caudal vertebrae into a pygostyle. Whether there were one or multiple mutations it should be noted that of the 37 posterior vertebral body-fusion mutants we examined [see Additional file 3], all but four are known to be caused by single mutational events, and have substantial phenotypic alterations not just to the tail, but to other parts of the skeleton as well. On the other hand, it should also be noted that most of the mutants that were considered for this study have mutations within gene coding regions. Mutations in CREs would likely have resulted in fewer pleiotropic effects in both mice and early birds. The nature of potential pleiotropic effects, however, should still be considered when addressing the issue of evolutionary morphological change. In this case, the pleiotropic effects of vertebral body mutations mirror a number of alterations observed in early birds, and these additional alterations occurred in the same timeframe as the transition to truncated tails. Since these pleiotropic effects (at least in the mouse) include fused vertebrae, not just in the tail but also in more anterior regions, the more substantial synsacral fusions observed in confuciusornithids, enantiornithines, and ornithurines could have been facilitated by a vertebral body mutation (or convergently by a similar mutation). Additional rib or uncinate process fusion, or even digit fusion, could also have occurred, which together with the other bone fusion and tail truncation phenotypes, could account for the relatively sudden appearance of these short-tailed birds in the fossil record. *Jeholornis* and *Confuciusornis* were likely contemporaries [156], and *Jeholornis* exhibited flight structures very similar to *Confuciusornis* but differed considerably in the posterior half of its body [157]. If a vertebral fusion mutation occurred in a primitive bird like *Jeholornis*, which fused additional vertebrae in its synsacrum, truncated its tail, and fused some ribs, the resulting creature would have come a long way towards resembling *Confuciusornis*. Once the mutation(s) had occurred, it/they were likely fixed in the population by the advantages conferred on flight and possibly on sexual selection display.

If we were to conjecture what was a likely type of mutation that occurred in a feathered maniraptoran dinosaur on its way to becoming a bird, based on the mouse mutant data, we would hypothesize that one or more mutations modulated genes involved in axial extension. Any number of axial extension genes could have been affected, but in the mouse, most mutations causing distally fused caudal vertebrae and shortened tails lie in the Notch/Wnt pathway, in somite segmentation, differentiation or somite boundary formation. Future comparative studies of signaling cascades between birds, long-tailed reptiles and mice should help to uncover these long-lost mutations, and further our understanding of the evolution of birds from non-avian maniraptoran dinosaurs.

## Abbreviations

AER: apical ectodermal ridge; BMP: bone morphogenetic protein; CML: caudofemoralis muscle, large; CNH: chordoneural hinge; CREs: *cis* regulatory regions; E: embryonic day; FGF: fibroblast growth factor; HH: Hamburger Hamilton; M: mesenchyme; Nc: notochord; NT: neural tube; PSM: presomitic mesoderm; RA: retinoic acid; SCD: spondylocostal dystosis; Shh: sonic hedgehog; TG: tailgut; VER: ventral ectodermal ridge.

## Competing interests

The authors declare that they have no competing interests.

## Authors’ contributions

DR performed the mouse tail mutant analysis, assembled the developmental axial extension and termination sections, designed Figures 2, 4 and 5, and was primarily responsible for writing the manuscript. SCC was responsible for the Araucana studies, Figures 7 and 8, and contributed to the writing of the manuscript. HCEL wrote the introduction and contributed to the section on bird tail development, and with DR, established the plan for the review. CO contributed to the introduction, and performed the analyses and subsequent execution of Figures 1 and 3. AB performed RA inhibition studies on chick embryo tails. CM is a co-PI for the overall project, and contributed to writing the manuscript. RB contributed to the mouse tail mutant analysis and to the review layout. JH is the PI, and spearheaded this project from its onset. JH provided details of the prevailing evolutionary theories discussed, and contributed to the project's direction. All authors critically edited the manuscript and approved it for submission.

## Authors’ information

DR is a post-doctoral fellow at the Museum of the Rockies, Montana State University, Bozeman, MT. SCC is an Associate Professor in the Department of Biological Sciences, Clemson University, Clemson, SC. HCEL is an Associate Professor at the Redpath Museum, McGill University, Montreal, Canada. CO is a Visiting Professor in the Department of Earth Sciences, Montana State University. AB is a Senior Research Assistant at Vaccine and Gene Therapy Florida, Port Lucie, FL; she was a former post-doctoral fellow at the Museum of the Rockies, Montana State University. CM is an Associate Professor in the Cell Biology and Neuroscience Department at Montana State University. RB is an Associate Professor in the Cell Biology and Neuroscience Department at Montana State University. JH is a Regents' Professor of Paleontology in the Department of Earth Sciences, Montana State University and is Curator of Paleontology at the Museum of the Rockies, Montana State University.

## Supplementary Material

Additional file 1 Excel spreadsheet of mouse tail mutants (16 pages). Click here for file

Additional file 2 **Abbreviations and references for Additional files ****1****and ****3****.** Click here for file

Additional file 3 Table of posterior vertebral body fusion mouse mutants. Click here for file
