# Supplementary material for: From dinosaurs to birds: a tail of evolution
Source: EvoDevo. 2014 Jul 29;5:25. doi: 10.1186/2041-9139-5-25 (PMC4304130; doi:10.1186/2041-9139-5-25)
Supplement: Additional file 2 — Abbreviations and references for Additional files 1and 3. [file 2041-9139-5-25-S2.pdf]

Additional file 2. Abbreviations and references for Additional Files 1 and 3.

I. Additional File 1

A. Abbreviations:

#: number of
*: restricted to
C: cervical
cdl: caudal
chng'd: changed
chrom: chromosome
cyto: cytochrome
dec: decreased
def: defect(s)
dep: dependent
disorg: disorganized
h-box: homeobox
hm: homozygote
hom: homeotic
ht: heterozygote
inc: increased
L: lumbar
n. d.: not determined
noto.: notochord
NT: neural tube
red: reduced
S: sacral
skel: skeletal
T: thoracic
transform: transformation
transx: transcription
v: vertebra(e)

B. References

Information presented in Additional File 1 was gleaned from the MGD mouse mutant database:

Eppig JT, Blake JA, Bult CJ, Kadin JA, Richardson JE: **The Mouse Genome Database Group. 2012. The Mouse Genome Database (MGD): comprehensive resource for genetics and genomics of the laboratory mouse.** *Nucleic acids research* 2012, **40:**D881-886.

Other noted references within the spreadsheet include:

1. Young T, Rowland JE, van de Ven C, Bialecka M, Novoa A, Carapuco M, van Nes J, de Graaff W, Duluc I, Freund JN, et al: **Cdx and Hox genes differentially regulate posterior axial growth in mammalian embryos.** *Developmental cell* 2009, **17:**516-526
2. Boulet AM, Capecchi MR: **Signaling by FGF4 and FGF8 is required for axial elongation of the mouse embryo.** *Dev Biol* 2012, **371:**235-245.

II. Additional File 3

A. Abbreviations

#: number of
*: restricted
cdl: caudal
chrom: chromosome
Dec.: decreased
dev: development
Dev.: Developmental
L: lumbar
N. Tube: Neural Tube
N-chord: notochord
reg: regulated
S: sacral
T: thoracic

B. References

1. Kaestner KH, Bleckmann SC, Monaghan AP, Schlondorff J, Mincheva A, Lichter P, Schutz G: **Clustered arrangement of winged helix genes fkh-6 and MFH-1: possible implications for mesoderm development.** *Development* 1996, **122:**1751-1758.
2. Avellino R, Carrella S, Pirozzi M, Risolino M, Salierno FG, Franco P, Stoppelli P, Verde P, Banfi S, Conte I: **miR-204 targeting of Ankrd13A controls both mesenchymal neural crest and lens cell migration.** *PloS one* 2013, **8:**e61099.
3. Jensen LD, Cao Z, Nakamura M, Yang Y, Brautigam L, Andersson P, Zhang Y, Wahlberg E, Lanne T, Hosaka K, Cao Y: **Opposing effects of circadian clock genes bmal1 and period2 in regulation of VEGF-dependent angiogenesis in developing zebrafish.** *Cell reports* 2012, **2:**231-241.
4. Jena N, Martin-Seisdedos C, McCue P, Croce CM: **BMP7 null mutation in mice: developmental defects in skeleton, kidney, and eye.** *Experimental cell research* 1997, **230:**28-37.
5. Young T, Rowland JE, van de Ven C, Bialecka M, Novoa A, Carapuco M, van Nes J, de Graaff W, Duluc I, Freund JN, et al: **Cdx and Hox genes differentially regulate posterior axial growth in mammalian embryos.** *Developmental cell* 2009, **17:**516-526.
6. McIntyre RE, Lakshminarasimhan Chavali P, Ismail O, Carragher DM, Sanchez-Andrade G, Forment JV, Fu B, Del Castillo Velasco-Herrera M, Edwards A, van der Weyden L, et al: **Disruption of mouse Cenpj, a regulator of centriole biogenesis, phenocopies Seckel syndrome.** *PLoS genetics* 2012, **8:**e1003022.
7. Lopez TP, Fan CM: **Dynamic CREB family activity drives segmentation and posterior polarity specification in mammalian somitogenesis.** *Proceedings of the National Academy of Sciences of the United States of America* 2013, **110:**E2019-2027.
8. Bafico A, Liu G, Yaniv A, Gazit A, Aaronson SA: **Novel mechanism of Wnt signalling inhibition mediated by Dickkopf-1 interaction with LRP6/Arrow.** *Nature cell biology* 2001, **3:**683-686.
9. Shinkai Y, Tsuji T, Kawamoto Y, Kunieda T: **New mutant mouse with skeletal deformities caused by mutation in delta like 3 (Dll3) gene.** *Experimental animals / Japanese Association for Laboratory Animal Science* 2004, **53:**129-136.
10. Wahl MB, Deng C, Lewandoski M, Pourquie O: **FGF signaling acts upstream of the NOTCH and WNT signaling pathways to control segmentation clock oscillations in mouse somitogenesis.** *Development* 2007, **134:**4033-4041.
11. Wong RL, Wlodarczyk BJ, Min KS, Scott ML, Kartiko S, Yu W, Merriweather MY, Vogel P, Zambrowicz BP, Finnell RH: **Mouse Fkbp8 activity is required to inhibit cell death and establish dorso-ventral patterning in the posterior neural tube.** *Human molecular genetics* 2008, **17:**587-601.
12. Plummer NW, Spicher K, Malphurs J, Akiyama H, Abramowitz J, Nurnberg B, Birnbaumer L: **Development of the mammalian axial skeleton requires signaling through the Galpha(i) subfamily of heterotrimeric G**

proteins. *Proceedings of the National Academy of Sciences of the United States of America* 2012, **109:**21366-21371.
13. Bessho Y, Hirata H, Masamizu Y, Kageyama R: **Periodic repression by the bHLH factor Hes7 is an essential mechanism for the somite segmentation clock.** *Genes & development* 2003, **17:**1451-1456.
14. Hu Y: **Abnormal Morphogenesis But Intact IKK Activation in Mice Lacking the IKK Subunit of IB Kinase.** *Science* 1999, **284:**316-320.
15. Okano S, Asano A, Sasaki N, Kon Y, Watanabe T, Agui T: **Examination of the Lunatic fringe and Uncx4.1 expression by whole-mount in situ hybridization in the embryo of the CKH-Jsr (jumbled spine and ribs) mouse.** *The Japanese journal of veterinary research* 2005, **52:**145-149.
16. Farkas DR, Chapman DL: **Kinked tail mutation results in notochord defects in heterozygotes and distal visceral endoderm defects in homozygotes.** *Dev Dyn* 2009, **238:**3237-3247.
17. Evrard YA, Lun Y, Aulehla A, Gan L, Johnson RL: **lunatic fringe is an essential mediator of somite segmentation and patterning.** *Nature* 1998, **394:**377-381.
18. Kokubu C, Heinzmann U, Kokubu T, Sakai N, Kubota T, Kawai M, Wahl MB, Galceran J, Grosschedl R, Ozono K, Imai K: **Skeletal defects in ringelschwanz mutant mice reveal that Lrp6 is required for proper somitogenesis and osteogenesis.** *Development* 2004, **131:**5469-5480.
19. Hollander WF, Waggie KS: **Meander tail: a recessive mutant located in chromosome 4 of the mouse.** *The Journal of heredity* 1977, **68:**403-406.
20. Mankoo BS, Skuntz S, Harrigan I, Grigorieva E, Candia A, Wright CV, Arnheiter H, Pachnis V: **The concerted action of Meox homeobox genes is required upstream of genetic pathways essential for the formation, patterning and differentiation of somites.** *Development* 2003, **130:**4655-4664.
21. Sasaki N, Kiso M, Kitagawa M, Saga Y: **The repression of Notch signaling occurs via the destabilization of mastermind-like 1 by Mesp2 and is essential for somitogenesis.** *Development* 2011, **138:**55-64.
22. Zhao X, D DA, Lim WK, Brahmachary M, Carro MS, Ludwig T, Cardo CC, Guillemot F, Aldape K, Califano A, et al: **The N-Myc-DLL3 cascade is suppressed by the ubiquitin ligase Huwe1 to inhibit proliferation and promote neurogenesis in the developing brain.** *Developmental cell* 2009, **17:**210-221.
23. Beckers A, Alten L, Viebahn C, Andre P, Gossler A: **The mouse homeobox gene Noto regulates node morphogenesis, notochordal ciliogenesis, and left right patterning.** *Proceedings of the National Academy of Sciences of the United States of America* 2007, **104:**15765-15770.
24. Kim W, Matsui T, Yamao M, Ishibashi M, Tamada K, Takumi T, Kohno K, Oba S, Ishii S, Sakumura Y, Bessho Y: **The period of the somite segmentation clock is sensitive to Notch activity.** *Molecular biology of the cell* 2011, **22:**3541-3549.
25. Schubert FR, Tremblay P, Mansouri A, Faisst AM, Kammandel B, Lumsden A, Gruss P, Dietrich S: **Early mesodermal phenotypes in splotch suggest a role for Pax3 in the formation of epithelial somites.** *Dev Dyn* 2001, **222:**506-521.

26. Zhang Y, Singh MK, Degenhardt KR, Lu MM, Bennett J, Yoshida Y, Epstein JA: **Tie2Cre-mediated inactivation of plexinD1 results in congenital heart, vascular and skeletal defects.** *Dev Biol* 2009, **325:**82-93.
27. Mazalouskas MD, Godoy-Ruiz R, Weber DJ, Zimmer DB, Honkanen RE, Wadzinski BE: **Small G Proteins Rac1 and Ras Regulate Serine/Threonine Protein Phosphatase 5 (PP5).Extracellular Signal-Regulated Kinase (ERK) Complexes Involved in the Feedback Regulation of Raf1.** *The Journal of biological chemistry* 2014, **289:**4219-4232.
28. Delfini MC, Dubrulle J, Malapert P, Chal J, Pourquie O: **Control of the segmentation process by graded MAPK/ERK activation in the chick embryo.** *Proceedings of the National Academy of Sciences of the United States of America* 2005, **102:**11343-11348.
29. Schwabe GC, Trepczik B, Suring K, Brieske N, Tucker AS, Sharpe PT, Minami Y, Mundlos S: **Ror2 knockout mouse as a model for the developmental pathology of autosomal recessive Robinow syndrome.** *Dev Dyn* 2004, **229:**400-410.
30. Kondrashov N, Pusic A, Stumpf CR, Shimizu K, Hsieh AC, Xue S, Ishijima J, Shiroishi T, Barna M: **Ribosome-mediated specificity in Hox mRNA translation and vertebrate tissue patterning.** *Cell* 2011, **145:**383-397.
31. Watkins-Chow DE, Cooke J, Pidsley R, Edwards A, Slotkin R, Leeds KE, Mullen R, Baxter LL, Campbell TG, Salzer MC, et al: **Mutation of the diamond-blackfan anemia gene Rps7 in mouse results in morphological and neuroanatomical phenotypes.** *PLoS genetics* 2013, **9:**e1003094.
32. Hegde S, Lenox LE, Lariviere A, Porayette P, Perry JM, Yon M, Paulson RF: **An intronic sequence mutated in flexed-tail mice regulates splicing of Smad5.** *Mammalian genome : official journal of the International Mammalian Genome Society* 2007, **18:**852-860.
33. Ratzka A, Kalus I, Moser M, Dierks T, Mundlos S, Vortkamp A: **Redundant function of the heparan sulfate 6-O-endosulfatases Sulf1 and Sulf2 during skeletal development.** *Dev Dyn* 2008, **237:**339-353.
34. White PH: **Defective somite patterning in mouse embryos with reduced levels of Tbx6.** *Development* 2003, **130:**1681-1690.
35. Biris KK, Dunty WC, Jr., Yamaguchi TP: **Mouse Ripply2 is downstream of Wnt3a and is dynamically expressed during somitogenesis.** *Dev Dyn* 2007, **236:**3167-3172.
36. Gao B, Song H, Bishop K, Elliot G, Garrett L, English MA, Andre P, Robinson J, Sood R, Minami Y, et al: **Wnt signaling gradients establish planar cell polarity by inducing Vangl2 phosphorylation through Ror2.** *Developmental cell* 2011, **20:**163-176.
37. Gofflot F, Hall M, Morriss-Kay GM: **Genetic patterning of the developing mouse tail at the time of posterior neuropore closure.** *Dev Dyn* 1997, **210:**431-445.
