# Supplementary material for: From dinosaurs to birds: a tail of evolution
Source: EvoDevo. 2014 Jul 29;5:25. doi: 10.1186/2041-9139-5-25 (PMC4304130; doi:10.1186/2041-9139-5-25)
Supplement: Additional file 3 — Table of posterior vertebral body fusion mouse mutants. [file 2041-9139-5-25-S3.pdf]

## Additional file 3. Posterior Vertebral Body Fusion Mouse Mutants

| Mutant | Affected Vertebrae | Dec. # cdl v | Fused Ribs | Fused Digits | Dev. Structure Affected | | | Viable | Fertile | Relevant Functions and Pathways |
|---|---|---|---|---|---|---|---|---|---|---|
| | | | | | Somite | N.Tube | N-chord | | | |
| *am amputated*<br>possible mutation: Foxc2<br>Spontaneous, recessive | data not shown | +++ | ✓ | | ✓ | | | — | | Foxc2 is involved in somite formation; tied to Shh & Bmp [1] |
| *Ankrd13a*<br>Gene trapped | cdl | | | | | | | ✓ | ✓ | cell-cell adhesion, also involved in neural crest [2] |
| *Arntl* (Bmal1 or MOP3)<br>Targeted knock-out | T, S | | | | | | | ✓* | ✓ | circadian clock gene tied to Notch-reg. angiogenesis [3] |
| *Bmp7*<br>Random gene disruption by insertion | T | | ✓ | | | | | — | | specifies ventroposterior fates and paraxial mesoderm [4] |
| *$Cdx2^{+/-}/Cdx4^{-/-}$*<br>Targeted knock-outs | S | +++ | | | ✓ | ✓ | | ✓ | ✓ | Hox pathway genes involved in posterior axial elongation; tied to Wnt and Fgf [5] |
| *Cenpj*<br>Targeted insertion | cdl | | | ✓ | | | | ✓ | ✓ | involved in centriole bio-genesis & chrom. stability [6] |
| *CREB*<br>Dominant negative knock-in | C, T, L, cdl | | ✓ | | ✓ | | | — | | Modulator of Notch/Wnt in somite segmentation & polarity establishment [7] |
| *$Dkk1^{d/-}$*<br>doubleridge/null compound | L, S, cdl | | | ✓ | ✓ | | | ✓ | ✓ | Lrp6 binding partner; modulates Wnt expression [8] |
| *Dll3*<br>Spontaneous or radiation-induced; recessive | C, T, L, S, cdl | ++ | ✓ | | ✓ | | | ✓ | ✓ | Notch/Wnt crosstalker during somitogenesis [9] |
| *Fgf3*<br>Targeted knock-out | cdl | ++ | | | ✓ | ✓ | | ✓* | ✓ | Upstream modulator of Notch/Wnt during somite polarity establishment [10] |
| *Fkbp8*<br>Gene trapped | T, L, S | | | | | ✓ | | ✓ | ✓ | Neural tube patterning; tied to Notch via Zic1 down-reg. [11] |

| | | | | | | | | | | |
|---|---|---|---|---|---|---|---|---|---|---|
| *Gnai3*<br>Targeted knock-out | L | data not shown | ✓ | | ✓ | | | ✓ | ✓ | tied to Bmp7, and Wnt/axin signaling [12] |
| *Hes7*<br>Targeted insertion knock-in | C, T, L, S, cdl | ++ | ✓ | | ✓ | | | ✓* | ✓ | Notch/Fgf crosstalker in somite segmentation clock [13] |
| *Ikkα*<br>Targeted reporter insertion | C, S, cdl | + | | ✓ | | | | — | | involved in epidermal cell-cell adhesion; facilitates bone formation via bFGF [14] |
| *Jsr* jumbled spine & ribs<br>Spontaneous, dominant | T, cdl | ++ | ✓ | | ✓ | | | ✓ | ✓ | mutation unknown; tied to Notch pathway via Uncx [15] |
| *Knk* kinked tail<br>Spontaneous, semi-dominant | cdl | + | | | | | ✓ | ✓ | ✓ | mutation unknown [16] |
| *Lfng*<br>Chemically induced single point mutation | T, L, S | ++ | ✓ | | ✓ | | | ✓* | ✓ | Notch pathway; involved in somite segmentation clock [17] |
| *Lrp6*<br>Spontaneous, semi-dominant | cdl | ++ | ✓ | | ✓ | ✓ | | ✓* | ✓ | Wnt/Notch crosstalker in somite segmentation [18] |
| *mea* meander tail<br>Spontaneous, recessive | T, L, cdl +<br>S vertebral spines | ++ | | | | | | ✓ | ✓ | mutation unknown [19] |
| *Meox1/Meox2*<br>Targeted knock-outs | cdl | ++ | ✓ | | ✓ | | | — | | involved in somite patterning; induced by retinoic acid [20] |
| *Mesp2*<br>Targeted insertion knock-out | C; L transverse process fusion | ++ | | | ✓ | | | ✓* | ✓ | Notch pathway; involved in somite boundary formation [21] |
| *N-myc*<br>alternative splice conditional mutation | L | | | | | | ✓ | ✓ | ✓ | promotes neurogenesis via N-myc/Dll3/Notch cascade [22] |
| *Noto*<br>Spontaneous single point mutation, recessive | L, S, cdl | ++ | | | ✓ | ✓ | ✓ | ✓ | ✓ | involved in notochord extension & node; controlled by Wnt [23] |
| *Nrarp*<br>Targeted knock-out | T, L, S, cdl | + | ✓ | | ✓ | | | ✓ | ✓ | Notch/Wnt crosstalker in somite segmentation clock [24] |
| *Pax3* splotch$^{d}$<br>Spontaneous, semi-dominant | C, T, L, S | | ✓ | | ✓ | ✓ | | — | | involved in preaxial meso-derm condensation for somitogenesis; tied toNotch pathway genes [25] |
| *Plxnd1*<br>Targeted knock-out | T, L | data not shown | ✓ | | | | | — | | Notch-controlled role in inter-somitic vascular development [26] |

| | | | | | | | | | | |
|---|---|---|---|---|---|---|---|---|---|---|
| *Ppp5c*<br>Targeted insertion | cdl | | | | | | | ✓ | ✓ | regulates ERK signaling [27]; ERK signaling is involved in Wnt/FGF controlled somite segmentation [28] |
| *rh* racchiterata<br>Spontaneous, recessive | L, S | + | ✓ | | ✓ | | | ✓ | ✓ | mutation unknown; tied to Shh via Pax1 [25] |
| *Ror2*<br>Targeted insertion knock-in | cdl | + | ✓ | ✓ | ✓ | | | ✓ | ✓ | Wnt Rc; involved in maintenance of presomitic mesoderm; also tied to Notch via Dll1, Lfng, & Mesp2 down-reg. [29] |
| *Rpl38*<br>Spontaneous, semi-dominant | C, T, cdl | ++ | ✓ | | | ✓ | | ✓* | ✓ | ribosomal protein involved in Hox gene control [30] |
| *Rps7*<br>Chemically induced single point mutation, dominant | cdl; S<br>transverse process fusion | + | | | | | | ✓* | ✓ | ribosomal protein; role in posterior dev. not studied [31] |
| *Sfxn1* f, flexed tail<br>Spontaneous, recessive | L, S, cdl | +/- | ✓ | | | ✓ | ✓ | ✓ | ✓ | Smad5/Bmp4 signaling defects [32] |
| *Sulf1/Sulf2*<br>Targeted insertion knock-outs | T, L, S, cdl | | | | ✓ | | | ✓ | ✓ | regulate growth factors; tied to Bmp & Shh signaling [33] |
| *Tbx6*<br>Spontaneous, duplication/ insertion | C; T, L trans-verse process fusion | + | ✓ | | ✓ | ✓ | ✓ | ✓ | ✓* | Notch-controlled role in somite boundary formation [34] |
| *Uncx*<br>Targeted reporter insertion | data not shown | | ✓ | | ✓ | | | — | | Notch signaling modulator in somite condensation and differentiation [35] |
| *Vangl2* (homozygote)<br>Spontaneous, single point mutation, semi-dominant | T, L, S, cdl | ++ | ✓ | | | ✓ | | — | | involved in convergent extension & cell polarity; tied to Wnt [36] |
| *Wnt5a*<br>Targeted insertion knock-out | S, cdl | +++ | | | ✓ | ✓ | | — | | Wnt secreted by the VER [37]; strain-dep. vertebral fusion & viability defects |
